# Sexual and asexual reproduction in a Mediterranean *Tethya* (Porifera, Demospongiae) species

**DOI:** 10.1186/s40850-022-00142-9

**Published:** 2022-07-22

**Authors:** Maria Mastrodonato, Giovanni Scillitani, Roberta Trani, Frine Cardone, Giuseppe Corriero, Carlotta Nonnis Marzano

**Affiliations:** 1 Department of Biology, University of Bari Aldo Moro, Via Orabona 4, 70125 Bari, Italy; 2 Consorzio Nazionale Interuniversitario per le Scienze del Mare (CoNISMa), 00196 Rome, Italy; 3 Department of Integrative Marine Ecology, Zoological Station Anton Dohrn, 80121 Naples, Italy

**Keywords:** Reproductive cycle, *Tethya*, Mediterranean Sea, Buds, Spermatic cysts

## Abstract

**Background:**

The reproductive cycle of the recently described sponge *Tethya meloni* was investigated for a period of 15 months (September 2018 – November 2019) in the Mar Piccolo of Taranto (Southern Italy) and was compared with data previously collected for the other two sympatric species of the same genus known for Mediterranean Sea, *T. citrina* and *T. aurantium.*

**Results:**

*T. meloni* is a gonochoric species with a sex ratio strongly shifted towards females. Asexual budding was a seasonal process, limited to few specimens. In a specimen collected in September 2018 both oocytes and buds occurred, suggesting that in *T. meloni* the sexual and asexual phases may coexist both at the population and individual levels.

**Conclusions:**

The data obtained from this research compared with the available literature confirm the high temporal variability of the reproductive cycles in the Mediterranean species of *Tethya*, but with common general characteristics. In sexual reproduction, the oocyte production period lasts several months, with a peak between summer and autumn while spermatogenesis, shorter but with greater reproductive effort, follows the onset of oogenesis. The asexual reproduction phase of *T. meloni*, on the other hand, occurs in a short period and seems to have less importance in the overall reproductive process.

## Background

Many sponges reproduce both sexually and asexually. In a number of marine species asexual reproduction involves the formation of buds, i.e. small functional bodies that develop on the external sponge surface and acquire autonomous life after the detachment from the parent. This strategy, occasionally described in some species of demosponges and homoscleromorphs [1–4] seems to be the rule in the families Polymastiidae (order Polymastiida), Tethyidae (order Tethyida) and Tetillidae (order Tetractinellida) [5–7]. In particular, in the genus *Tethya,* a cosmopolitan group of demosponges encompassing more than 150 species [8], the production of such asexual bodies may involve a strong effort in terms of resource allocation [6, 9].

The reproductive activity has been widely studied in the Mediterranean *T. aurantium* and *T. citrina*, from different points of view [6, 9–15]. In both species, budding has been described both as a seasonal process [6] and a continuous event, with a short seasonal decrease [9], thus suggesting the role of environmental factors in affecting the asexual reproductive pattern. In both cases, however, the two species exhibit a large involvement of resources toward asexual reproduction, with a massive bud production [9]. As regards sexual reproduction, literature data indicate that both species are oviparous and gonochoric [6, 15] and show a similar cycle, with a summer period of oocyte production, widely overlapping in the two species, although *T. citrina* seems to mature earlier. Males are very rare possibly due to the very short period of spermatogenesis [6, 11].

*T. aurantium* and *T. citrina* have long been considered the only species representing the genus *Tethya* in the Mediterranean. Recently, based on several records in different geographic areas, Corriero et al. (2015) suggested to add a third species, *T. meloni*, to the Mediterranean list. Since Mediterranean *Tethya* species are morphologically very similar and often have a sympatric distribution [16–18] it is interesting to investigate whether their reproductive patterns differ.

**Fig. 1:**
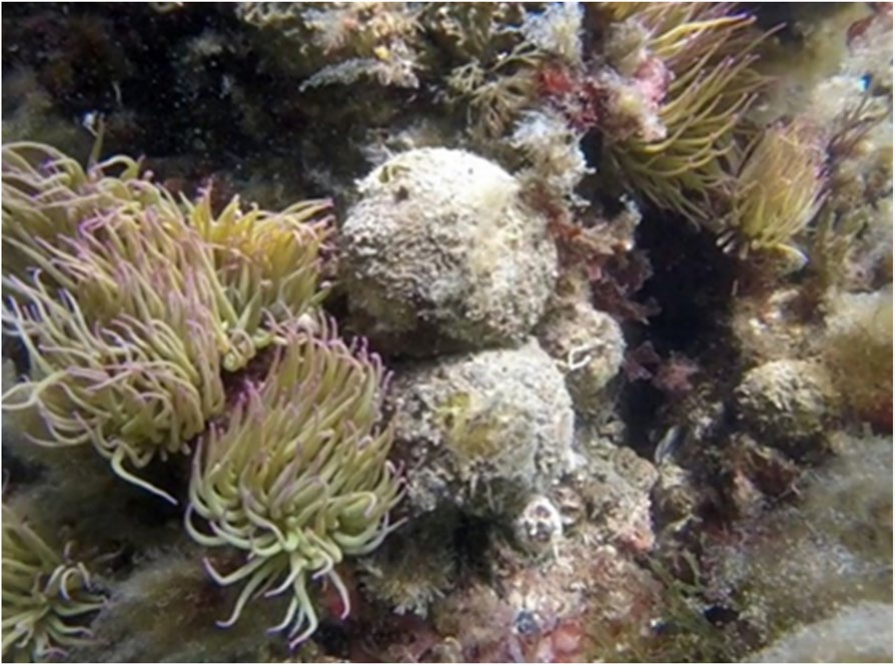
*Tethya meloni.* Specimens at a depth of 2 m in the Mar Piccolo of Taranto (Southern Italy). Photo credit: Roberta Trani

Thus, we studied the reproductive cycle of a population of *T. meloni* (Fig. 1) with the aim of providing information on the relative importance of the sexual and asexual phases in the life cycle of this known species.

## Results

### Taxonomic analysis of the collected specimens

The analysis of the sponge specimens showed that morphological and skeletal traits overlap with those reported for *Tethya meloni* [16] in all the 169 sampled specimens (Table 1). In agreement with the literature data, *T. meloni* resembles the sympatric *T. aurantium* for the large body size and the well-developed cortex. In both species, the external and thick cortex is characterized by large and flattened tubercles. In comparison with *T. citrina*, *T. meloni* shows similarities in the colour of the surface (grey to yellow vs cream, respectively) and in some spicular traits. However, oxyspherasters in *T. meloni* are larger (65.7–119 μm in diameter; mean value: 106.5 μm) (Table 1) compared to *T. citrina* (18–64.8 μm; mean value: 42.9 μm) [19]. In both species oxyspherasters are characterized by long rays, with highest values of *R/C* (ray length/centre diameter) index in *T. meloni* (1–1.8), with respect to *T. citrina* (0.6–1.4) [19].

**Table 1:** *Tethya meloni.* Spicule size in the studied specimens

Strongyloxeas min-max length (μm)	Strongyloxeas min-max width (μm)	Oxyspherasters (mean diameter ± *SD*; μm)	Oxyspherasters (mean *R/C* ± *SD*)	Cortical micrasters (mean *R/C* ± *SD*)	Choanosomal micrasters (mean *R/C* ± *SD*)
365–1710	7–35	106.5 ± 15.3	1.2 ± 0.3	14.1 ± 2.1	15.4 ± 2.6

*SD* standard deviation, *R* ray length, *C* centre diameter

### Sexual reproduction

**Fig. 2:**
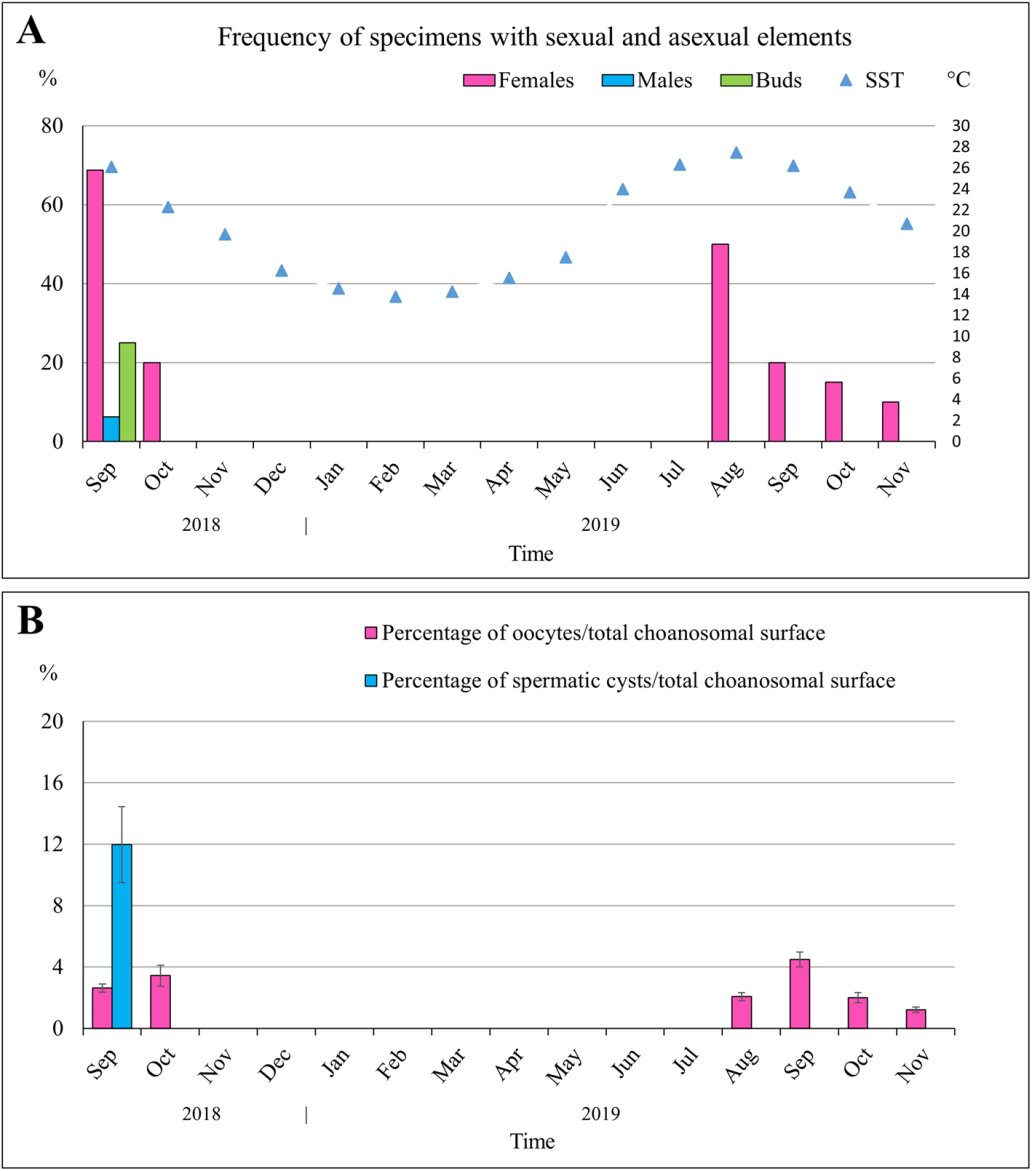
*Tethya meloni.* Reproductive effort at the Mar Piccolo of Taranto during the study period. **A** Frequency of specimens of *T. meloni* with sexual reproductive elements and buds. The secondary axis on the right refers to mean monthly values of sea surface temperature, *SST*. **B** Percentage of sponge mesohyl occupied by oocytes and spermatic cysts. Bars indicate standard errors

**Fig. 3:**
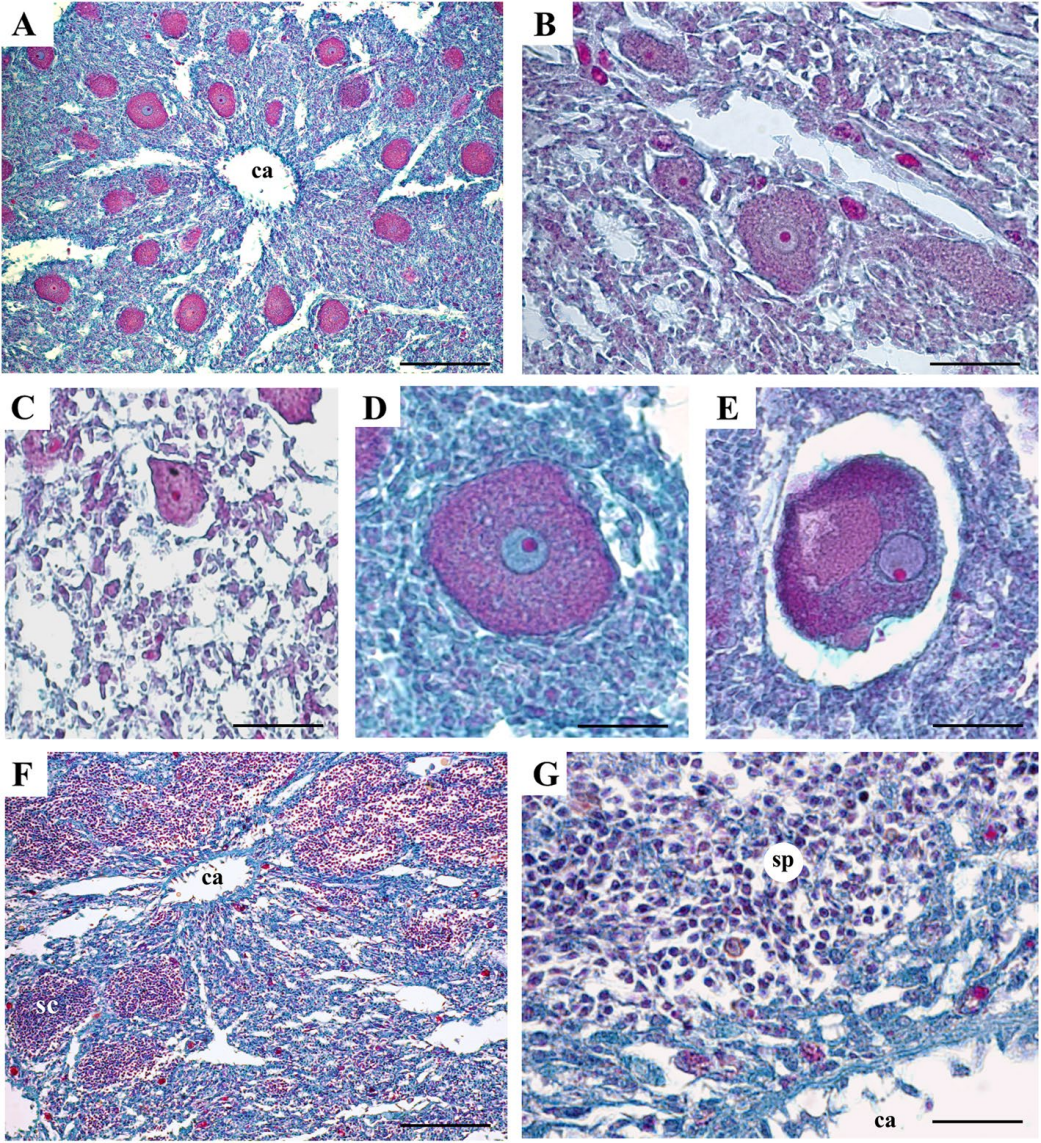
*Tethya meloni*. Histological sections: **A, B** Clusters of young oocytes, in (**A)** they surround a canal (ca) of the sponge aquiferous system. **C-E** oocytes at different maturation stages. **F, G** spermatic cysts (sc) and spermatocytes (sp) around the canals (ca) of the aquiferous system. Mallory’s trichrome stain. Space bars: **A, F** = 100 μm; **B** = 50 μm; **C**-**E, G** = 25 μm

A total of 34 specimens with sexual reproductive elements was found over 169 examined specimens (20.1%). During 2018, oocytes were detected in September and October (Fig. 2A), showing in October the largest average dimensions (more than 60 μm in diameter) (Table 2). In 2019, young oocytes appeared in August and were still present in November. At the onset of oogenesis, they measured 20–30 μm in diameter (Table 2) and appeared round in shape, forming numerous clusters within the sponge choanosome (Fig. 3A-C). Mature oocytes, about 50 μm in diameter, were detected in October and November 2019 and were characterized by a cytoplasm filled with inclusions and a nucleolated nucleus measuring about 13 μm in diameter (Fig. 3D, E).

**Table 2:** *Tethya meloni.* Size of sexual reproductive elements (oocytes and spermatic cysts)

		Oocytes (diameter, μm)	Spermatic cysts (major axis length, μm)
2018	September	38.1 ± 0.8	122.0 ± 7.7
	October	64.2 ± 1.4	–
2019	August	27.3 ± 3.9	–
	September	34.8 ± 1.0	–
	October	48.37 ± 7.9	–
	November	53.0 ± 1.3	–

During the study period, the frequency of specimens with oocytes was highest in September 2018 and in August 2019 (69 and 50%, respectively), then it progressively dropped (Fig. 2A). The sponges constantly devoted a small part of their choanosome to the production of gametes, as the percentage of choanosome occupied by the oocytes never exceeded 5% (Fig. 2B).

Comparing the two reproductive periods, no significant differences were found for the reproductive effort of females between 2018 and 2019 (K-W test: *H-stat*_*1df*_ = 1.609, *p* = 0.204). The maximum percentage of choanosome occupied by oocytes (4.5%) occurred in September 2019 (Fig. 2).

Spermatic cysts were detected only in September 2018, in a single specimen which however showed a rather high percentage of choanosome occupied by cysts (12%, Fig. 2). They were delimited by pinacocytes (Fig. 3F, G) and exhibited an elliptic form, with the major axis ranging between 74.4 and 172.0 μm (mean value 122.0 ± 7.7; Table 2).

The trend of monthly average *SST* relative to the study site and period is reported in Fig. 2. Overall, the thermal regime is highly variable, with Winter (January–March) minima ranging from 13.2 to 13.4 °C and Summer (July–September) maxima from 27.4 to 28.9 °C. Both the frequency of specimens with oocytes and the percentage of sponge choanosome occupied by oocytes seem to correspond to the trend of monthly *SST* (Spearman’s *ρ* = 0.828, *p* = 0.04) (Fig. 2).

### Asexual reproduction

**Fig. 4:**
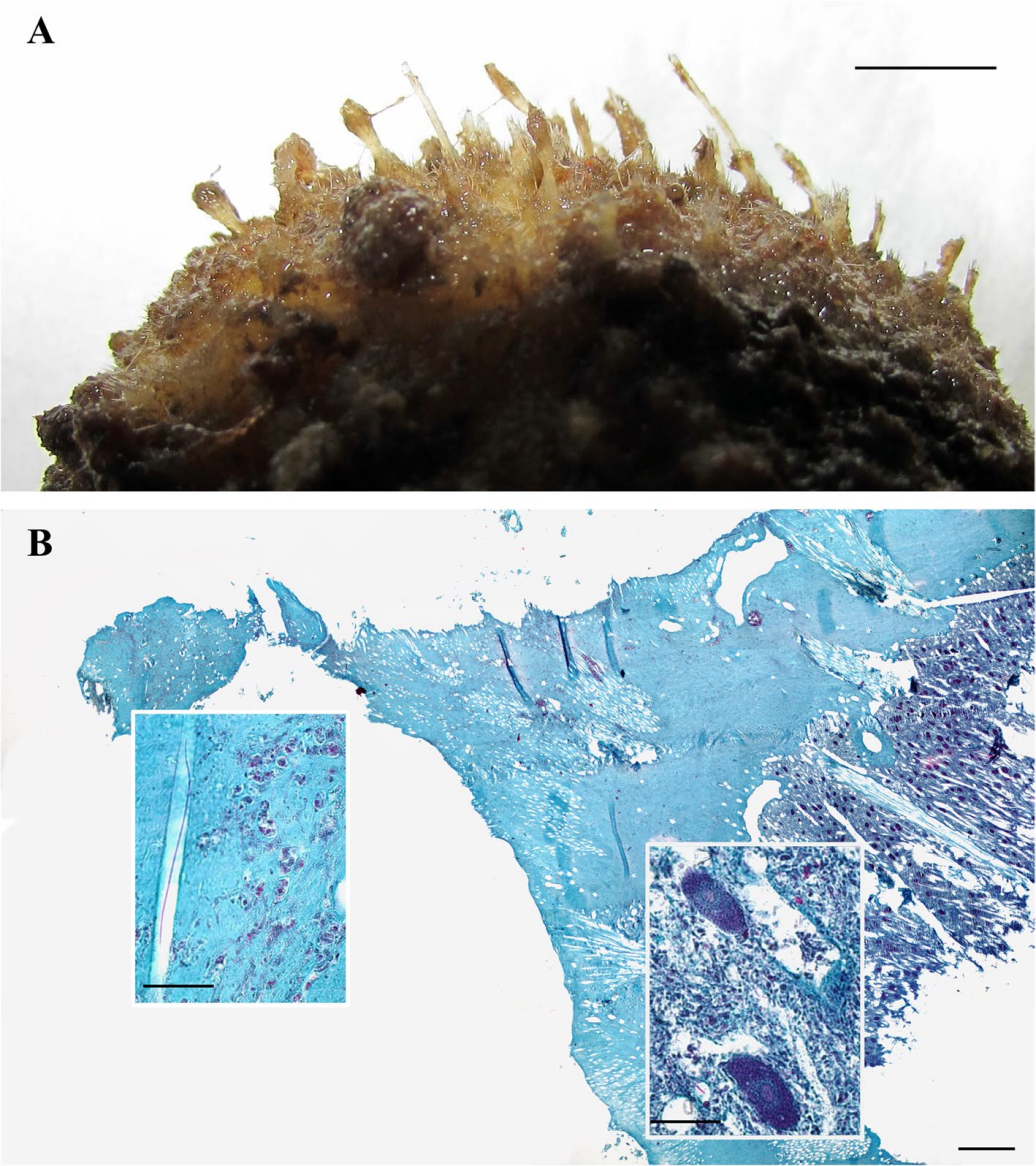
*Tethya meloni.* Specimen with simultaneous presence of asexual and sexual elements. **A** Buds protruding from the sponge cortical surface. Space bar: 5 mm. **B** Histological section showing the occurrence of a bud (on the left) and some oocytes (on the right) in the same specimen. Left insert: detail of the bud. Right insert: oocytes in the choanosome of the parent sponge. Mallory’s trichrome stain. Space bars: main = 500 μm; inserts = 50 μm

Asexual elements were only detected in September 2018, when about 25% of the sponge specimens (2.4% of the total specimens collected) showed the presence of buds (Fig. 2B). The mean number of buds per specimen was 55 ± 8.9. Buds sprout out from the sponge surface as spherical small bodies connected to the parent by a stalk (Fig. 4A). Their diameter varied from 0.9 to 1.1 mm. Histological analysis revealed the lack of choanocyte chambers also in largest buds, together with a scarce differentiation between cortical and choanosomal layers (Fig. 4B) and the occurrence of several sparse cells with inclusions.

## Discussion

Literature data about the sexual reproductive cycle of *Tethya aurantium* and *T. citrina* in Mediterranean Sea show a certain degree of variability, even at intraspecific level. In *T. aurantium* oocyte production occurred from August to November and spermatic cysts from August to October, whereas oogenesis was delayed of about one month in *T. citrina* [11]. In both species, males were very rare, with 6 specimens over 863 examined in *T. aurantium* and 6 over 522 in *T. citrina* [11]. No further males in these two species were recorded in other research. Oocytes, on the contrary, were repeatedly observed from July to August in about 150 examined specimens of *T. aurantium* [15]. In north-western Sicily the production of female elements extended from May to September in *T. aurantium* and from April to August in *T. citrina* [6].

As regards asexual reproduction, in the lagoon of Marsala buds occurs throughout the year with an Autumn-Winter peak in *T. aurantium*, and only in Autumn and Winter in *T. citrina* [6]. This trend markedly differed from that reported for *T. citrina* from the Mar Piccolo of Taranto, living sympatrically with the studied population of *T. meloni*, where a continuous bud production was observed during a two-year study. In particular, peaks in frequency values (100%) were recorded in September and October and from February to April, when density values increased up to 40 buds/cm^2^ of sponge external surface, suggesting a key role of asexual reproduction for the maintenance and dispersal of *T. citrina* in this lagoon environment [9].

Few data are available in the literature about the reproduction in *T. meloni*, since this species has been only recently described [16]. The results of the present research indicate that sexual and asexual reproductive activities coexist, not only in the same population but also in the same specimen of *T. meloni*. Therefore, unlike what has been observed in other demosponges [6, 20], in the studied species the reproductive effort can be directed to both the sexual and asexual phases, suggesting that the allocation of resources do not act competitively.

As regards sexual reproduction, the population of *T. meloni* from the Mar Piccolo of Taranto proved to be gonochoric and oviparous, coherently with all other species of *Tethya* studied to date, both in the Mediterranean and elsewhere (see [21]). The present research highlights a strong periodicity in the production of gametes, which is concentrated in Summer and early Autumn and is mainly devoted to the oogenesis, with a single male found in September 2018. The *sex ratio* strongly oriented towards females is a common feature among sponges (e.g. in [1]), with the congeneric *T. burtoni* from New Zealand showing only one male out of 995 sponge specimens collected over a two-year study [21]. Sexual reproduction appears to be linked to the trend of monthly water temperature recorded in the Mar Piccolo of Taranto during the study period, similarly to what observed in other studies regarding sponge reproduction. Indeed, water temperature is usually considered one of the main environmental factors influencing reproduction in marine sponges, with increases and decreases in temperature often coinciding with the onset of reproduction.

In fact, for a number of sponges including *Geodia cydonium* [22] and *Tethya citrina* [9] gamete production and budding have been shown to occur during periods of warmer temperatures. By contrast, decreasing temperatures are correlated with reproductive and budding events for other sponges, such as for example *Mycale contarenii* [1].

About 2% of budding specimens were found in populations of *T. meloni* from different Mediterranean habitats and geographic areas [16], and also in the population object of the present study the value recorded is low. As observed in other *Tethya* species [10, 13, 23] the buds of *T. meloni* lack of differentiation between cortical and choanosomal layers. In addition, they lack of aquiferous system and choanocyte chambers. Thereafter, the differentiation of the aquiferous system in *Tethya* buds seems to occur after their detachment from the parent sponge [10]. On the other hand, the presence of choanocyte chambers has been described only in *Mycale* (*Aegagropila*) *contarenii* (Martens, 1824) [1, 24], *Oscarella lobulari*s (Schmidt, 1862) and *O. tubercolata* (Schmidt, 1868) [2], *Haliclona fulva* (Topsent, 1893) [4], *T. seychellensis* (Wright, 1881) [23], *T. wilhelma* Sarà, Nickel & Brümmer, 2001 [25] and in the freshwater *Radiospongilla cerebellata* (Bowerbank, 1863) [26]. The lack of the aquiferous system in buds hampers their water pumping, this latter being responsible for the uptake and transportation of nutrients in sponges. As suggested for buds of *T. aurantium* [23] cell inclusions observed in the asexual elements of *T. meloni* could represent stored material useful to sustain morphogenetic processes leading to the acquisition of a complete functionality.

## Conclusions

The data obtained from this research compared with the vast literature available confirm the high variability of the reproductive cycles in the Mediterranean species of *Tethya*. This despite the previous results on the reproduction of these latter [6, 9, 11, 15] were obtained from populations that could include specimens of the current *T. meloni*, described only later [16]. On the whole, it is possible to outline some general features: 1) prolonged period of oocyte production showing a Summer or Autumn peak and a moderate reproductive effort, with low percentages of choanosome occupied by oocytes; 2) short spermatogenesis after the start of the oocytes production, with a higher reproductive effort in the involved specimens with respect to the oogenesis process; 3) budding as usual asexual mode of reproduction in all three species. However, while *T. aurantium* and *T. citrina* employ considerable resources in the budding process, which shows a cyclical pattern throughout the year, in *T. meloni* the asexual phase occurs for a short period and seems to have less importance in the overall reproductive process.

## Methods

### Sampling area

Sponge samples were collected from the Mar Piccolo of Taranto, an inner sea located north of the Gulf of Taranto (Ionian Sea, SE Italy). This is a lagoon environment, characterized by a reduced water exchange with the open sea and by hydrological parameters showing a variable trend according to the season. In particular, salinity ranges from 34.3 to 37.7‰ [27], though this latter may locally drop down to 25–30‰, due to the continental inflow [9]. The sampling area (40°29′07″ N, 17°16′39″ E) is characterized by abundant, artificial hard substrates that host a rich and highly diversified macrozoobenthic filter-feeder assemblage, (poriferans, hydrozoans, polychaetes, bryozoans, bivalves, crinoids and ascidians). Sponges, in particular, include more than 30 species, that may locally reach high abundance values [28]. The occurrence of *Tethya aurantium*, *T. citrina* and *T. meloni* in the Mar Piccolo of Taranto has been repeatedly reported in the literature [16, 28].

### Experimental protocol

Along a 15 months period (September 2018–November 2019), about 10 specimens of *T. meloni* were monthly sampled by SCUBA divers at a depth of two meters in the inner area of the lagoon (Fig. 1), for a total of 169 specimens sampled. In the laboratory, all the sponges were photographed with a metric reference, the presence of any buds was recorded, and buds were counted. For taxonomic analysis, sponge spicule slides were made using standard procedures used for demosponges [29]. For each specimen, 20 spicules from each category were then measured at the light microscope.

For histological observations, sponge samples were fixed in 10% neutral buffered formalin. Then, a portion of each sample was desilicified by immersion in 5% hydrofluoric acid for one hour, dehydrated using a graded ethanol series and then embedded in paraffin wax (see [30] for details). Since gametes occur in the internal choanosomal layer only, the cortical tissue was eliminated before the inclusion. Serial sections, 5 μm thick, were cut using a rotary microtome (Leica RM 2155, Leica, Wetzlar, Germany). Rehydrated sections were stained using Mallory’s trichrome method for distinguishing cellular from extracellular components. Collagen fibres stained an intense blue, cytoplasm stained reddish, and nucleoli stained pinkish. Images were captured in bright light using an Eclipse E600 photomicroscope and a DMX1200 digital camera (Nikon Instruments SpA, Calenzano, Italy).

In order to estimate the reproductive effort of *T. meloni*, we analysed digital photographs of ten microscopic sections, for a total surface of 0.3 mm^2^. Thus, we determined the number of specimens with sexual reproductive elements and estimated number and area (%) of oocytes and spermatic cysts within the sponge choanosome using ImageJ Software. Reproductive effort was expressed as percentage of reproductive tissue (mean ± *SD*) in the sponges and related to local values of sea surface temperature (*SST*, °C). These latter were supplied by Copernicus Marine Service (https://marine.copernicus.eu) as daily temperatures, subsequently transformed into monthly average temperatures.

### Statistical analysis

The difference between the reproductive effort in the two years (2018 and 2019) was assessed with a Kruskal–Wallis (K-W) nonparametric rank-sum test (*H*). Since September and October were the only months for which data were available in both years, we limited statistical comparison to them. The correlation between reproductive effort and sea surface temperature (*SST*) was tested by computing a Spearman’s rank correlation coefficient between monthly percentage of reproductive tissue and monthly coefficients of variation calculated from mean and standard deviation values of *SST*. The data analysis was generated using the Real Statistics Resource Pack software Release 7.6 [31].

## Data Availability

Data and materials are available from Roberta Trani on reasonable request.

## Abbreviations

*C*: Centre diameter of oxyspheraster
ca: Canals
*df*: Degrees of freedom
*H-stat*: Statistics computed for the Kruskal-Wallis test
K-W: Kruskal-Wallis test
*p*: Probability
*ρ*: Spearman’s rank correlation coefficient
*R*: Ray length of oxyspheraster
*R/C*: Oxyspheraster ray length/centre diameter index
*SD*: Standard deviation
SE: South East
*SST*: Sea surface temperature
sc: Spermatic cysts
sp.: Spermatocytes
